# The effects of single high-dose or daily low-dosage oral colecalciferol treatment on vitamin D levels and muscle strength in postmenopausal women

**DOI:** 10.1186/s12902-018-0277-8

**Published:** 2018-07-21

**Authors:** Mahmut Apaydin, Asli Gencay Can, Muhammed Kizilgul, Selvihan Beysel, Seyfullah Kan, Mustafa Caliskan, Taner Demirci, Ozgur Ozcelik, Mustafa Ozbek, Erman Cakal

**Affiliations:** 1 Department of Endocrinology and Metabolism, Diskapi Training and Research Hospital, Ankara, Turkey; 2 Department of Physical Medicine and Rehabilitation, Diskapi Training and Research Hospital, Ankara, Turkey

**Keywords:** Vitamin D, Muscle strength, Postmenopausal women

## Abstract

**Introduction:**

Vitamin D deficiency is a common health problem. Vitamin D supplements are used to improve vitamin D status; however, there are contradictory data related to what doses to give and how often they should be given. Many studies have investigated the effects of vitamin D supplementation on muscle strength, but the results remain controversial. We aimed to compare the effects and safety of single high-dose with daily low-dose oral colecalciferol on 25(OH)D levels and muscle strength in postmenopausal women with vitamin D deficiency or insufficiency.

**Methods and design:**

Sixty healthy postmenopausal women who had serum vitamin D levels < 20 ng/mL (50 nmol/L) were enrolled in the study. Group 1 (*n* = 32) was given daily oral dosages of 800 IU vitamin D3, and group 2 (*n* = 28) was given a single oral dose of 300,000 IU vitamin D3. Serum vitamin D levels and muscle strengths were measured at the beginning, 4th, and 12th week. Muscle strength tests were performed at 60° using a Biodex system 3 isokinetic dynamometer.

**Results:**

Pretreatment vitamin D levels did not differ between the two groups (10.2 ± 4.4 ng/mL (25,4 ± 10,9 nmol/L); 9.7 ± 4.4 ng/mL (24,2 ± 10,9 nmol/L), *p* > 0.05). A significant increase in vitamin D levels was observed in both groups at 4 and 12 weeks after vitamin D3 treatment. The increase in the single-dose group was significantly higher than the daily low-dosage group at the 4th week (35.9 ± 9.6 ng/mL (89,6 ± 23,9 nmol/L), 16.9 ± 5.8 ng/mL (42,1 ± 14,4 nmol/L), *p* = 0.01). The increase in the single-dose group was significantly higher than in the daily low dosage group at the 12th week (23.4 ± 4.7 ng/mL (58,4 ± 11,7 nmol/L), 19.8 ± 7.2 ng/mL (49,4 ± 17,9 nmol/L), *p* = 0.049). The quadriceps muscle strength score increased significantly in the daily group at the 4th week (*p* = 0.038). The hamstring muscle strength score increased significantly in the daily group at the 12th week (*p* = 0.037).

**Conclusion:**

Although daily administration routes are more effective in improving muscle strength, a single administration is more effective in increasing vitamin D levels.

**Trial registration number:**

ISRCTN14226530 (04.07.2018), Name of the registry: ISRCTN registry, The study was retrospectively registered.

## Background

Vitamin D is a fat-soluble vitamin that is now known to play an important role in a variety of biologic functions including immune regulation, proliferation, differentiation, apoptosis, and angiogenesis, in addition to being the main hormone regulating calcium phosphate homeostasis and mineral bone metabolism [1]. Vitamin D deficiency is often undiagnosed and untreated because it has insidious or nonspecific signs and symptoms, and is a common health problem worldwide [2]. Many authors agree that a 25-hydroxyvitamin D3 (25(OH)D3) concentration less than 20 ng/mL (50 nmol/L) is defined as vitamin D deficiency; however, a 25(OH)D3 concentration between 21 and 29 ng/mL (50–74 nmol/L) is defined as vitamin D insufficiency [3]. The form of vitamin D therapy, the dose and dosing interval, and route of administration are not given much importance because there are no specific recommendations or guidelines in this regard [4]. It is well documented that increasing 25(OH)D3 serum levels in patients with vitamin D deficiency increases intestinal calcium absorption, decreases parathyroid hormone (PTH) levels, fall incidence and fracture risk, and increases muscle strength [5]. Serum 25(OH)D3 is accepted as the best indicator of vitamin D condition [6]. The cell nuclei of muscle cells express the vitamin D receptor (VDR) [7] and vitamin D has an impact on muscle cell contractility [8]. Vitamin D deficiency leads to disruption in muscular protein synthesis and consequently muscle mass, and finally muscle strength decreases as a result [9]. Vitamin D deficiency may lead to myopathy. Vitamin D supplementation is considered to have an influence on muscle fiber composition and morphology in vitamin D deficient older adults [10].

In a study conducted in 167 postmenopausal women, it was demonstrated that a serum 25(OH)D3 level higher than 20 ng/mL (50 nmol/L) was achieved with a vitamin D(3) dosage of 800 IU/d in 97.5% of women [11]. It is known that daily vitamin D supplementation reduces the frequency of falls and fractures in older women [12]. However, poor adherence to oral vitamin D replacement is a common clinical problem [13, 14]. Several studies about the pharmacokinetics, biochemical effects, efficacy, and safety proved that a single large dose of colecalciferol was safe, well tolerated, and effective [15–19]. In a systematic review of studies using large, single-dose, oral vitamin D supplementation in adult populations, a single vitamin D3 dose of ≥100,000 IU was shown to provide a perdurable effective means of increasing short-term vitamin D concentrations to > 20 ng/mL (50 nmol/L). However, larger vitamin D3 doses of ≥300,000 IU were required to achieve 25(OH)D3 concentrations > 30 ng/mL (75 nmol/L) and decreased plasma PTH concentrations [20]. Despite the presence of many studies on this topic, an accepted recommendation about the best dose and dose interval is still lacking.

Many studies have investigated the effects of vitamin D supplementation on muscle function but the results remain controversial. Some studies observed a favorable effect of vitamin D supplementation on muscle strength [21, 22], whereas others failed to show this beneficial effect [16, 23, 24]. A meta-analysis demonstrated that muscle strength could be improved by vitamin D supplementation [25]. On the contrary, another meta-analysis including 12 studies, which focused on older subjects with baseline 25(OH)D3 concentrations higher than (10 ng/mL) 25 nmol/L, indicated no beneficial effect of vitamin D supplementation on muscle strength [26].

The primary aim of this study was to evaluate and compare the effects and safety of single high- dose with daily low-dosage oral colecalciferol on 25(OH)D3 levels in older patients with vitamin D deficiency or insufficiency.

## Methods

We enrolled 60 healthy, postmenopausal women aged 50–68 years whose vitamin D level was lower than 20 ng/mL (50 nmol/L). The participants, who were followed up by our clinic in Diskapi Training and Research Hospital, were consecutively randomized to the administration of daily 800 IU (*n* = 32) or a single oral bolus of 300,000 IU (*n* = 28) of vitamin D_3_. Individuals who had granulomatous conditions, thyroid disease, malabsorption syndromes, liver disease, kidney disease, diabetes or postural instability (cerebellar disease, vestibular disease, vitamin B12 deficiency, drugs), and individuals taking anticonvulsants, calcium or vitamin D supplements, barbiturates, or steroids in any form were excluded. The study protocol was approved by the medical ethics committee of the Ankara Diskapi Training and Research Hospital, and all subjects gave written informed consent.

### Design overview

Our study was a randomized clinical trial that lasted 3 months. The participants were collected in the spring and winter of 2015–2016 to minimize seasonal effects. Serum vitamin D levels and muscle strengths were measured at the beginning, and 4th and 12th weeks of treatment. The physiotherapist who assessed all functional endpoints was blinded to both regimens and the physiotherapist who performed the assessments was blinded to the treatment groups.

### Analytical methods

Calcium and phosphate were measured using an enzyme method with an autoanalyzer (ADVIA 2400, Siemens Healthcare Diagnostics Inc., Tarrytown, NY, USA). iPTH and 25(OH)D were measured using an immunochemiluminescent assay (Siemens Advia Centaur XP, Siemens Healthcare Diagnostics Inc., Tarrytown, NY, USA).

Quadriceps (knee extensors) and hamstring (knee flexors) muscle strengths of each leg were measured in Biodex System 3 isokinetic dynamometer (Biodex, Shirley, NY) at the beginning, and 4th and 12th weeks of treatment. The patients were strapped into the chair with their knee flexed at 90°. The anatomic axis of the knee (lateral femoral condyle) was aligned with the axis of the dynamometer. The resistance pad was placed proximal to the medial malleolus. A seat belt harness was placed around the patient’s chest and thigh for stabilization. The range of motion varied from 90° knee flexion to full extension. Maximal quadriceps and hamstring peak torques (Nm) were obtained through concentric isokinetic knee extension and flexion performed at the angular velocity of 60°/sn for 5 consecutive contractions. The highest peak torque value of these 5 contractions was recorded. The value of peak torque for each muscle was divided by body weight and the relative peak value (Nm/kg) was calculated. Isokinetic muscle strength was assessed using the relative peak value. All patients indicated that their right leg was dominant.

### Statistical analysis

Statistical analysis was performed using the SPSS 18.0 (SPSS, Inc) software. Descriptive analyses are expressed as the mean ± standard deviation (SD) and percentages (%). Normality was tested using the Kolmogorov-Smirnov and Shapiro-Wilk W tests. The Chi-square test or Fisher’s exact test, where appropriate, were used to compare categorical variables. Student’s t-test was used for normally distributed continuous variables. The Mann-Whitney U test was used for continuous variables that were not normally distributed. The paired samples t-test was used for normally distributed continuous variables. *p* <  0.05 was considered as statistically significant.

## Results

The clinical characteristics of study subjects are summarized in Table 1. There was no significant difference between the daily dosage and single-dose group according to age, body weight, height, and body mass index (BMI) (*p* > 0.05). There was no significant difference between the two groups in terms of sun exposure, physical activity, milk consumption, smoking habits, and clothing style (*p* > 0.05).

**Table 1:** The clinical characteristics of the daily dosage group and the single-dose group

	Daily dosage group (*n* = 32)	Single-dose group (*n* = 28)	*p* value
Characteristics (mean ± SD)			
Age (years)	51.60 ± 5.80	51.58 ± 5.54	0.644
Height (cm)	160.40 ± 7.94	158.75 ± 5.10	0.152
Body weight, pretreatment (kg)	76.51 ± 12.51	72.04 ± 12.34	0.115
BMI, pretreatment (kg/m^2^)	29.75 ± 5.31	28.61 ± 4.96	0.354
Body weight, 12th week, (kg)	74.51 ± 12.51	72.48 ± 12.84	0.544
BMI, 12th week, (kg/m^2^)	29.07 ± 5.34	28.8 ± 5.24	0.855
Sun exposure (%)			0.255
< 1 h/d	0	3.6	
1–2 h/d	15.6	17.9	
2–4 h/d	56.3	39.3	
4–7 h/d	21.9	38.5	
> 7 h/d	6.3	0	
Physical activity (%)			0.377
< 1 h/d	0	3.6	
1–2 h/d	15.6	14.3	
2–4 h/d	50.0	39.3	
4–7 h/d	28.1	42.9	
> 7 h/d	6.3	0	
Milk consumption (%)			0.306
< 1 portion/d	21.9	22.2	
1–2 portion/d	46.9	63.0	
2–4 portion/d	31.3	14.8	
Smoking habits (%)			0.179
No	71.9	71.4	
Quit	15.6	3.6	
Yes	12.5	25.0	
Clothing style (%)			0.727
Western-style clothing	37.5	39.3	
Traditional	37.5	28.6	
Oriental-style clothing	25.0	32.1	

There was no difference in serum glucose, total cholesterol, low-density lipoprotein (LDL)-cholesterol, triglycerides, high-sensitivity C-reactive protein (hs-CRP) and thyroid-stimulating hormone (TSH) levels between the single-dose and daily-dosage groups at pretreatment, 4th, and 12th week (*p* > 0.05). Serum calcium and alkaline phosphatase and PTH levels were not different between the single-dose and daily-dosage groups at pretreatment, 4th, and 12th week (p > 0.05). The single-dose (9.7 ± 4.4; 35.9 ± 9.6 and 23.1 ± 4.7 ng/mL (24.2 ± 10.9; 89.6 ± 23.9 and 57.6 ± 11.7 nmol/L), *p* <  0.001) and daily-dosage groups (10.2 ± 4.4, 16.9 ± 5.8, and 19.8 ± 7.2 ng/mL(25.4 ± 10.9; 42.1 ± 14.4 and 49.4 ± 17.9 nmol/L), *p* <  0.001) had lower pretreatment vitamin D levels than in the 4th and 12th week, respectively. Pretreatment vitamin D levels did not differ between the groups (*p* > 0.05). The vitamin D level was higher in the single-dose group than in the daily-dosage group at the 4th (*p* = 0.001) and 12th week (*p* = 0.49). The daily-dose group had lower pretreatment phosphorus levels than in 4th and 12th week (*p* <  0.001), whereas the single-dose group had higher phosphorus levels at the 4th week than at pretreatment and the 12th week (*p* = 0.043). Phosphorus levels were not different between two groups at pretreatment, 4th, and 12th week (*p* > 0.05). Pretreatment magnesium levels were not different between the two groups (*p* > 0.05). The pretreatment osteocalcin level was higher in the single-dose group than in the daily-dosage group (*p* = 0.022). The osteocalcin level was not different between the two groups at the 4th and 12th week (*p* > 0.05). The biochemical results of the single-dose and daily-dosage group at pretreatment, 4th, and 12th week are shown in Table 2.

**Table 2:** The biochemical results of the single-dose and daily-dosage group at pretreatment, 4th, and 12th week

	Single-dose group				Daily-dosage group			
	Pretreatment	4th week	12th week	*p*	Pretreatment	4th week	12th week	*p*
Glucose (mg/dL)	88.2 ± 9.8	81.7 ± 17.4	92.4 ± 9.5	0.014	89.1 ± 7.5	85.3 ± 6.8	88.6 ± 18.0	0.016
Total cholesterol (mg/dL)	209.5 ± 37.4	209.1 ± 27.0	204.7 ± 35.4	0.569	203.6 ± 36.6	1999.1 ± 38.8	212.3 ± 33.6	0.763
Triglycerides (mg/dL)	136.5 ± 49.6	115.6 ± 43.7	128.7 ± 56.1	0.108	148.5 ± 72.7	146.6 ± 90.3	154.6 ± 61.9	0.601
LDL-cholesterol (mg/dL)	133.4 ± 32.3	137.5 ± 23.7	131.3 ± 26.7	0.411	125.6 ± 27.8	122.1 ± 31.1	134.5 ± 32.2	0.301
Calcium								
(mg/dL)	9.5 ± 0.4	9.5 ± 0.4	9.4 ± 0.5	0.507	9.4 ± 0.4	9.5 ± 0.5	9.6 ± 0.4	0.059
(mmol/L)	2.3 ± 0.1	2.3 ± 0.1	2.3 ± 0.1		2.3 ± 0.1	2.3 ± 0.1	2.4 ± 0.1	
Phosphorus (mg/dL)	3.5 ± 0.4	3.8 ± 0.4	3.6 ± 0.6	0.043	3.4 ± 0.5	3.9 ± 0.5	3.9 ± 0.5	< 0.001
Magnesium (mg/dL)	2.1 ± 0.2				2.2 ± 0.2			
iPTH (pg/ml)	55.1 ± 24.3	47.7 ± 19.1	50.7 ± 20.5	0.152	62.4 ± 24.9	55.9 ± 22.8	54.9 ± 20.6	0.081
Vitamin D								
(ng/mL)	9.7 ± 4.4	35.9 ± 9.6	23.1 ± 4.7	< 0.001	10.2 ± 4.4	16.9 ± 5.8	19.8 ± 7.2	< 0.001
(nmol/L)	24.2 ± 10.9	89.6 ± 23.9	57.6 ± 11.7		25.4 ± 10.9	42.1 ± 14.4	49.4 ± 17.9	
Osteocalcin (ng/ml)	21.2 ± 19.1	21.9 ± 7.5	20.5 ± 7.6	0.231	11.2 ± 8.3	22.0 ± 7.2	20.4 ± 5.5	0.008
Alkaline phosphatase (units/L)	72.5 ± 20.7	73.0 ± 23.1	73.6 ± 19.8	0.138	69.6 ± 17.6	68.1 ± 19.6	68.0 ± 19.1	0.051
TSH (mIU/L)	2.5 ± 1.5	2.8 ± 2.0	2.4 ± 1.5	0.580	2.2 ± 1.1	2.2 ± 1.3	2.2 ± 1.4	0.965
hs-CRP (mg/L)	3.3 ± 3.1	3.2 ± 3.2	2.4 ± 2.2	0.188	4.5 ± 4.9	4.1 ± 3.7	4.1 ± 3.9	0.376

The relative isokinetic peak values for knee extensor and flexor (both dominant and non-dominant) muscles did not differ between pretreatment, 4th, and 12th week in the single-dose group (*p* > 0.05). The pretreatment peak values for dominant knee extensors were lower as compared with the 4th week in the daily-dosage group (0.97 ± 0.4 vs. 1.06 ± 0.4 N/m/kg, respectively, *p* = 0.03). Pretreatment peak values for non-dominant knee flexors were lower as compared with the 12th week in daily-dosage group (0.32 ± 0.1 vs. 0.37 ± 0.1 Nm/kg, respectively, p = 0.03). The daily-dosage group had similar dominant knee flexors and non-dominant knee extensors between pretreatment, 4th, and 12th week (*p* > 0.05). The relative peak values for knee extensor and flexor (both dominant and non-dominant) muscles did not differ between the daily- dosage and single-dose group according to pretreatment, 4th, and 12th week (*p* > 0.05). The relative isokinetic peak values of the dominant and non-dominant knee extensor and flexor muscles are shown in Table 3. Muscle function tests measured at 1st month in daily dose group were similar between patients who achieved 25OHVitD_3_ level of > 20 ng/mL (50 nmol/L) and patients with lower than 20 ng/mL (50 nmol/L).

**Table 3:** Relative isokinetic peak torques (Nm/kg) of the knee extensor and flexor muscles

	Knee extension					Knee flexion				
	Daily-dosage group		Single-dose group		*p*-value^a^	Daily -dosage group		Single-dose group		*p*-value^a^
	Dominant	Non-dominant	Dominant	Non-dominant		Dominant	Non-dominant	Dominant	Non-dominant	
Pretreatment	0.97 ± 0.4	0.98 ± 0.4	1.02 ± 0.3	0.99 ± 0.4	0.62/0.89	0.34 ± 0.1	0.32 ± 0.1	0.36 ± 0.2	0.33 ± 0.2	0.62/0.81
4th week	1.06 ± 0.4	1.09 ± 0.4	1.05 ± 0.3	1.06 ± 0.3	0.95/0.78	0.37 ± 0.2	0.36 ± 0.2	0.39 ± 0.2	0.39 ± 0.2	0.69/0.59
12th week	1.03 ± 0.3	1.05 ± 0.4	1.13 ± 0.3	1.14 ± 0.3	0.35/0.36	0.37 ± 0.1	0.37 ± 0.1	0.39 ± 0.2	0.40 ± 0.2	0.69/0.55
*p* value	0.03^b^	0.21	0.20	0.05		0.18	0.03^c^	0.22	0.10	

All patients in both groups had baseline vitamin D levels lower than 20 ng/mL (50 nmol/L). Serum 25-(OH)D levels greater than 20 ng/mL (50 nmol/L) were achieved in 96.7% of patients in the single large dose group at the end of 4th week, whereas it was 19.4% of patients in the daily low-dosage group (*p* < 0.001) (Table 4). Serum 25-(OH)D levels greater than 30 ng/mL (75 nmol/L) were achieved in 63.3% of patients in single large dose group at the end of 4th week compared with 3.2% in the daily low-dosage group (p < 0.001). Serum 25-(OH)D levels greater than 20 ng/mL (50 nmol/l) were achieved in 63.3% of patients in the single large dose group at the end of 12th week, whereas it was 45.2% in the daily low-dosage group (*p* > 0.05). The proportion of patients reaching vitamin D levels of 30 ng/mL (75 nmol/L) were similar in both groups (6.7% vs. 6.5%, p > 0.05).

**Table 4:** Percentage of patients that reach target serum 25 OH vitamin level based on treatment duration and replacement style

	Daily dose group	Single dose group	*p*
4th week Vitamin D < 20 Vitamin D ≥ 20	23 (% 74.2) 6 (% 19.4)	0 (% 0) 29 (% 96.7)	< 0.001
4th week Vitamin D < 30 Vitamin D ≥ 30	28 (% 90.3) 1 (% 3.2)	10 (33.3%) 19 (63.3%)	< 0.001
12th week Vitamin D < 20 Vitamin D ≥ 20	12 (38.7%) 14 (45,2%)	8 (26.7%) 19 (63.3%)	0.360
12th week Vitamin D < 30 Vitamin D ≥ 30	24 (77.4%) 2 (6.5%)	25 (83.3%) 2 (6.7%)	0.775

## Discussion

25(OH)D3 levels increased significantly in both groups at weeks 4 and 12 week after vitamin D treatment. The increase in the single-dose group was significantly higher than in the daily low- dosage group at the 4th and 12th week.

In a study, it was demonstrated that a serum 25(OH)D3 level higher than 20 ng/mL (50 nmol/L) was achieved with a vitamin D(3) dosage of 800 IU/d in 97.5% of women. Approximately 50% of patients in the same study group reached vitamin D levels of 30 ng/mL (75 nmol/L) [11]. In the present study, 45.2% of patients reached serum 25(OH)D3 levels of more than 20 ng/mL (50 nmol/L) with daily 800 IU vitamin D replacement dosages, whereas only 6.5% of patients reached serum 25(OH)D3 levels higher than 30 ng/mL (75 nmol/L). This difference may be partly explained by the difference between baseline vitamin D levels in both studies (10.2 ng/mL (25.4 nmol/L) vs. 15.6 ng/mL (38.6 nmol/L)).

Vitamin D levels significantly increased at the 4th week when compared with baseline in a study conducted with single high dose of vitamin D (300,000 IU); however, vitamin D levels at the 12th week were similar at baseline [4]. A recent study demonstrated that a single dose of 250,000 IU of vitamin D3 concluded in a robust increase in plasma 25(OH)D3 after 5 days, but it decreased to baseline levels after 90 days. The authors proposed that a larger dose or more frequent dosing regimen might be required for long-term management of vitamin D insufficiency [27]. In our study, vitamin D levels increased significantly in the single high-dose group at the 4th and 12th weeks after vitamin D3 treatment; however, vitamin D levels decreased at the 12th week when compared with the 4th week in the single large-dose group. Maintenance doses with regular intervals would be reasonable in patients undergoing single large-dose vitamin D replacement.

Human skeletal muscles express vitamin D receptors (VDR) and genotypic variations for this receptor have been reported to be related to decreased muscle strength. It is known that vitamin D has an important role in muscle strength and function. This condition is more prominent in proximal muscles of lower extremity. Because of this, we evaluated knee muscles strength such as quadriceps and hamstrings. It is difficult to evaluate the hip muscles strengh with isokinetic device, therefore we selected the knee muscles [28]. Moreover, vitamin D deficiency-associated muscle weakness is predominantly of the proximal muscle groups and has an effect on daily living activities including walking and climbing stairs [10].

Marantes et al. observed no consistent association between 25(OH)D3 levels and any measurements related to muscle mass or strength in either men or women. The authors proposed that factors affecting neuromuscular function rather than muscle strength might be responsible for the association between low 25(OH)D3 and increased fall risk observed in other studies [29]. Several studies evaluated lower leg isometric muscle strength [16, 19, 23, 30–34]; however, only two demonstrated an improvement in isometric muscle strength after treatment [19, 31]. Pfeifer et al. found significantly increased quadriceps strength after 6 months of treatment with 800 IU/ day vitamin D3 [33]. Similarly, Moreira-Pfrimer et al. demonstrated an improvement in maximal isometric strength of hip flexors and knee extensors in vitamin D3-treated subjects (150,000 IU once a month during the first 2 months, followed by 90,000 IU once a month for the last 4 months) [19]. Handgrip strength after vitamin D replacement was evaluated in five studies, and none was able to show significant effects [23, 30, 32, 35, 36]. A recent meta-analysis reported muscle strength measures of 29 randomized controlled trials involving 5533 subjects [37]. The results demonstrated that vitamin D replacement had a small, but significantly positive impact on global muscle strength. A significant positive effect on lower limb muscle strength was observed, but handgrip muscle strength was not affected. Supplementation seemed to be more effective in patients who presented with a 25(OH)D3 levels < 12 ng/mL (30 nmol/L) and aged 65 years or over. The authors proposed that these results could explain the significant effect of vitamin D on falls determined in meta-analyses [38, 39]. Indeed, quadriceps strength was determined to be a significant predictor of fall incidence [40]. We found a significant improvement in dominant quadriceps and non-dominant hamstring muscle strengths in the daily vitamin D group. Non-dominant quadriceps and dominant hamstring muscle strengths were also increased but the differences were not statistically significant. The sample size of our study was not large enough to detect a significant difference. The differences in dose of oral vitamin D, patient population, treatment interval, and muscle strength assessment test have been considered to be responsible for the inconsistencies.

In our study, both replacement types were found to be safe but vitamin D levels were demonstrated to be higher after replacement with single large doses. However, the effect of daily low-dosage vitamin D replacement on muscle strength was better than single large-dose replacement. These findings might support the results of a randomized controlled trial which demonstrated high-dose colecalciferol leads to a higher risk of falls and fractures in older community-dwelling women [41]. Long-term studies with larger populations investigating skeletal development, bone health maintenance, and non-skeletal effects of vitamin D are required to clarify the best replacement dose and form for adequate vitamin D levels in the maintenance of health.

Being a single center stduy and a relatively small sample size were the limitations of the study. Being at lower threshold levels of vitamin D_3_ which known to decrease fracture risk and falls at 1st and 3th of months of study could be another limitation of the study (referans), as it may make difficult to interpret the results of the study. Additionally, none of our patients was evaluated for vitamin D metabolites which has been demonstrated to be related to muscle function in recent studies. This is another limitation of our study.

## Conclusion

Although daily administration routes are more effective in improving muscle strength, a single administration is more effective in increasing vitamin D levels.

## Abbreviations

BMI: body mass index
CD: Celiac disease
DBP: diastolic blood pressure
EMA: anti endomysium antibody
ERS: endoplasmic reticulum stress
FPG: fasting plasma glucose
HC: hip circumference
HLD-C: HDL cholesterol
LDL-C: LDL-cholesterol
NIDDM: non-insulin dependent diabetes mellitus
PPPG: post-prandial plasma glucose
SBP: systolic blood pressure
T2DM: type 2 diabetes mellitus
TLR: Tool-like receptors
tTGA IgA: tissue transglutaminase antibody IgA
VA: villous atrophy
WC: waist circumference
